# Femto- to Millisecond Time-Resolved Photodynamics of a Double-Functionalized Push–Pull Organic Linker: Potential Candidate for Optoelectronically Active MOFs

**DOI:** 10.3390/ijms21124366

**Published:** 2020-06-19

**Authors:** Mario Gutiérrez, Lucie Duplouy-Armani, Lorenzo Angiolini, Mercedes Pintado-Sierra, Félix Sánchez, Abderrazzak Douhal

**Affiliations:** 1 Departamento de Química Física, Facultad de Ciencias del Medio Ambiente y Bioquímica and INAMOL, Universidad de Castilla-La Mancha, Avenida Carlos III, S/N, 45071 Toledo, Spain; Mario.Gutierrez@alu.uclm.es (M.G.); duplouy-armani.lucie@live.fr (L.D.-A.); lore.angiolini@gmail.com (L.A.); 2 LASIR—Laboratoire de Spectrochimie Infrarouge et Raman, CNRS, UMR 8516, University of Lille, F-59000 Lille, France; 3 Instituto de Química Orgánica, CSIC, Juan de la Cierva, 3, 28006 Madrid, Spain; m.pintado@csic.es (M.P.-S.); felix-iqo@iqog.csic.es (F.S.)

**Keywords:** intramolecular charge transfer, ultrafast spectroscopy, photochemistry, solvatochromism

## Abstract

The design of improved organic linkers for the further engineering of smarter metal–organic framework (MOF) materials has become a paramount task for a wide number of material scientists. In this report, a luminescent double-functionalized push–pull (electron donor–acceptor) archetype organic molecule, dimethyl 4-amino-8-cyanonaphthalene-2,6-dicarboxylate (Me_2_CANADC), has been synthesized and characterized. The optical steady-state properties of Me_2_CANADC are strongly influenced by the surrounding environment as a direct consequence of its strong charge transfer (CT) character. The relaxation from its first electronically excited singlet state follows a double pathway: (1) on one side deactivating from its local excited (LE) state in the sub-picosecond or picosecond time domain, and (2) on the other side undergoing an ultrafast intramolecular charge transfer (ICT) reaction that is slowing down in viscous solvents. The deactivation to the ground state of these species with CT character is the origin of the Me_2_CANADC luminescence, and they present solvent-dependent lifetime values ranging from 8 to 18 ns. The slow photodynamics of Me_2_CANADC unveils the coexistence of a non-emissive triplet excited state and the formation of a long-lived charge separated state (2 µs). These observations highlight the promising optical properties of Me_2_CANADC linker, opening a window for the design of new functional MOFs with huge potential to be applied in the fields of luminescent sensing and optoelectronics.

## 1. Introduction

The design and fabrication of smarter functional materials for real-world applications has become a priority for a large number of material researchers. Particularly, the field of porous crystalline metal–organic framework (MOF) materials has been extensively boosted during the last two decades [1]. This has been possible due to a combination of diverse factors, such as the chemical tunability of MOFs (exchangeability of metal clusters and organic linkers), which has favored the synthesis of an uncountable number of MOF materials, their excellent physicostructural conditions (ordered structures with large surface areas and accessible pores) and their promising abilities to be deployed into a wide range of technological applications [2,3,4,5]. In fact, MOFs and guest@MOF composites have emerged as versatile candidates in energy management [6,7], storage of fuels such as H_2_ and methane [8,9], and biological or medical applications [10,11,12]. Among all the possible MOF structures and composites, those who emit light have become very popular because of their possible integration in different photonic technologies as luminescent chemical and physical sensors or optoelectronic devices [13,14,15,16]. The luminescence properties of MOFs may arise from the organic linker, the metal clusters, or through internal interactions, such as ligand-to-metal, metal-to-ligand, ligand-to-ligand or metal-to-metal charge transfer interactions [17,18,19,20,21]. Based on that, a sophisticated approach for obtaining functional MOFs is through a well-planned design of the organic linkers [22,23,24,25]. Several examples can be found in the literature, where the linkers are the responsible for the active properties of MOFs either for luminescent applications or for other purposes, such as gas capture [22,23,24,25]. For example, it is very well known that the functionalization of the organic linkers with amine or diamine groups improves the CO_2_ adsorption capabilities of MOFs as a result of chemisorption mechanisms [24,25]. The smart construction of organic linkers is also the reason behind the very interesting luminescent properties exhibited by some MOFs [22,23]. For instance, two different carboxylate linker derivatives of 1,3,5-benzenetribenzoate were used in the fabrication of Zr-MOFs [23]. The functionalization of these linkers with methyl groups forces the peripheral rings to situate perpendicularly to the central one and at the same time confers an increase in the hydrophobicity of the materials, making them more stable in water. This fact, jointly to the linker emission properties, made possible the use of these MOFs for the detection of antibiotics and organic explosive molecules in water [23]. Another very interesting example is given by the stylish synthesis of an organic linker (H_2_tpdc-dpa) that contains electron donor and acceptor groups separated by a large dihedral angle [22]. The idea was to reduce the energy gap between the singlet and triplet excited states, favouring the process known as thermally activated delayed fluorescence (TADF). The aforementioned linker was used in the synthesis of a Zr-based MOF, which retains the TADF properties of the linker, having this special importance for its promising potential to be applied in the next generation of light-emitting diodes [26,27].

**Scheme 1:**
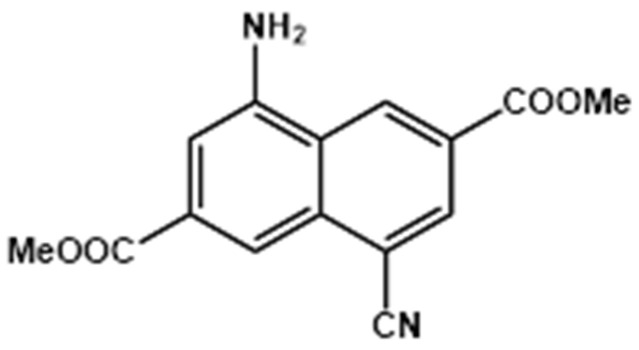
Molecular structure of Me_2_CANADC.

Even though a broad number of luminescent organic linkers have been used to synthesize emissive MOFs [21,28,29,30], to the best of our knowledge, there are not many examples in which the organic linkers are designed to fabricate electroluminescent MOFs. To overcome the lack of electroactive linkers, herein, we report on a double-functionalized linker, dimethyl 4-amino-8-cyanonaphthalene-2, 6-dicarboxylate (Me_2_CANADC, Scheme 1), in which the naphthalene central core is decorated with an electron donor (NH_2_) and acceptor (CN) pendant groups, leading to a push–pull archetype linker. It is very well-known that those push–pull systems undergo intramolecular charge transfer (ICT) reactions, which in many cases generate long-lived charge-separated states that are the keystone for their future implementation in optoelectronics or photocatalysis technologies [31,32,33]. We found that the double functionalization of Me_2_CANADC makes its photophysical properties dominated by a strong ICT character. The absorption and emission spectra are strongly influenced by the surrounding environment, specifically by the solvent polarity. For instance, the Stokes-shift values obtained for Me_2_CANADC in the different solvents have been well-correlated with their polarity function through the Lippert–Mataga equation, reflecting the involvement of an ICT phenomenon leading to a large change in the dipole moment upon electronic excitation (about 10 D). The emission lifetime of these species having an ICT character is also affected by the solvent polarity, showing values in the range of 8 to 18 ns. A deeper insight into the ultrafast photodynamics reveals that upon photoexcitation, two events are competing: the deactivation from the local excited (LE) state and an ultrafast ICT reaction, happening in tens of femtoseconds in non-protic solvents and slowed-down to tens of picoseconds in highly viscous solvents. The slow photodynamics was also investigated, and the involvement of a non-emissive triplet state (T_1_) as well as the formation of a long-lived charge separated (CS) state were noticed. Therefore, the double functionalization with electron donor and acceptor groups allows the synthesis of an optically active linker with promising and tuneable luminescence properties for the further fabrication of electroactive and luminescent-sensing MOF materials. Our results remark the importance of a well-planned design in the synthesis of functional organic linkers, paving the way for the engineering of new improved smarter materials.

## 2. Results and Discussion

### 2.1. Steady-state UV-Vis Absorption and Emission Studies

**Figure 1:**
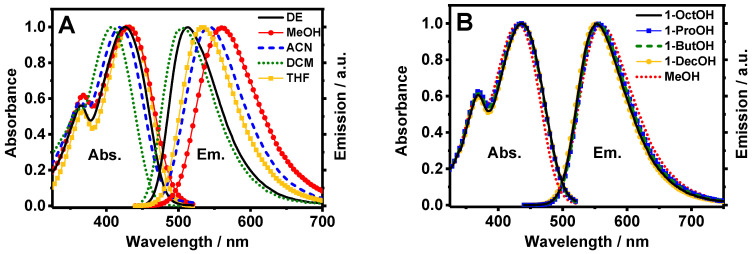
Normalized UV-visible absorption and fluorescence spectra of dimethyl 4-amino-8-cyanonaphthalene-2,6-dicarboxylate (Me_2_CANADC) linker in (**A**) dichloromethane (DCM) (green short dot), acetonitrile (ACN) (blue short dashes), diethyl ether (DE, black solid line), methanol (MeOH, red full circles), tetrahydrofuran (THF, orange full squares) and in (**B**) MeOH (red short dots), 1-propanol (1-ProOH, blue full squares), 1-butanol (1-ButOH, green short dashes), 1-octanol (1-OctOH, black solid line), and 1-decanol (1-DecOH, orange full circles). The excitation wavelength for the fluorescence spectra was 425 nm.

To begin with, the steady-state UV-visible absorption and emission properties of Me_2_CANADC in the selected organic solvents were explored. Figure 1A shows the absorption and emission spectra of Me_2_CANADC in diethyl ether (DE), dichloromethane (DCM), acetonitrile (ACN), tetrahydrofuran (THF), and methanol (MeOH) solutions. The shape of all absorption spectra is very similar independently of the used solvent, showing an intense broad band at lower energies and a second less intense hump appearing at higher energies (approximately 375 nm). However, the absorption spectra exhibit wavelength displacements with maxima ranging from 408 to 437 nm, depending on the solvent (Table 1). This phenomenon could be an indication of a certain degree of a charge transfer (CT) character of Me_2_CANADC in its ground state, as expected when the naphthalene core is functionalized with electron donor–acceptor (NH_2_–CN) pendant groups. Comparing the absorption of the unfunctionalized molecule (methyl 2,6-naphthalene dicarboxylate, Me_2_NDC), which is characterized by well-resolved vibrational bands with maximum at approximately 350 nm [18], and the absorption of the monofunctionalized 4-amino-2,6-naphthalenedicarboxylate (Me_2_NADC) in DCM solvent (broad band with maximum at approximately 390 nm) [19]; the simultaneous presence of electron donor and acceptor groups in Me_2_CANADC generates a bathochromic shift in its absorption spectrum of 58 nm and 18 nm (approximately 4060 and 1130 cm^−1^), respectively. The loss of the vibrational resolution alongside the bathochromic shift supports our previous attribution of the CT character of this push–pull Me_2_CANADC linker.

The emission spectrum of Me_2_CANADC upon excitation at 425 nm is characterized by a single broad band with maximum intensities ranging between 506 and 559 nm, depending on the solvent. Again, the shift in the emission spectrum with the solvent properties clearly evidences the influence of a CT character in the optical properties of Me_2_CANADC. To unveil how the optical properties of Me_2_CANADC are affected by its surrounding, the observed Stokes-shift value is evaluated considering the solvent orientation polarizability (∆*f*) as well as the viscosity (η) of the solvent (Table 1).

**Figure 2:**
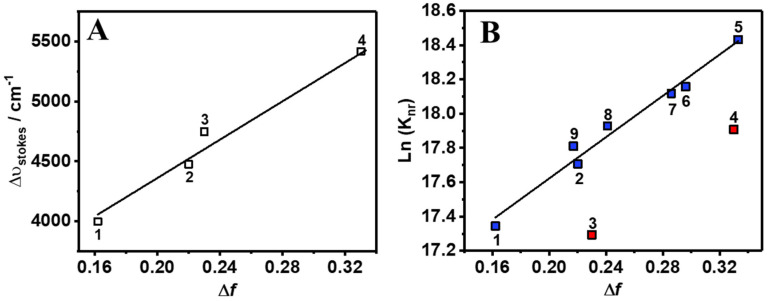
(**A**) Lippert–Mataga plot showing the dependence between the Stokes shift ($∆{\tilde{\nu }}_{Stokes}$) and solvent orientation polarizability (∆*f*), and (**B**) show the dependence of Ln(k_nr_) with ∆*f* of all the solvents except DCM (3) and ACN (4), which are shown as red squares. The labeling of the solvent is shown in Table 1. Graph (**A**) was lineally fitted to y = 8397x + 2683, with R^2^ = 0.97, *n* = 4, S.D. slope = 907 and S.D. (intercept) = 220. Graph (**B**) was lineally fitted to y = 6.01x + 16.4, with R^2^ = 0.97, *n* = 7, S.D. slope = 0.40 and S.D. (intercept) = 0.10.

Interestingly, the Stokes-shift value of Me_2_CANADC in different polar protic solvents having a similar polarity function (1-ProOH, 1-ButOH, and 1-OctOH), but growing viscosity, is almost unaffected (Figure 1B and Table 1), excluding the contribution of the viscosity on its steady-state optical solvatochromic properties. On the contrary, the Stokes-shift value in non-protic and methanol solvents exhibits a strong dependence with their orientation polarizability (∆*f*), having values ranging between 0.162 and 0.333 (Table 1). The Stokes-shift values and the orientation polarizability of the non-protic solvents have been correlated using the Lippert-Mataga equation [34,35,36,37]:

(1) $$∆{\tilde{\nu }}_{Stokes}={\tilde{\nu }}_{abs}-{\tilde{\nu }}_{em}=\frac{2}{hca_{0}^{3}}\left(\frac{\varepsilon_{s}-1}{2\varepsilon_{s}+1}-\frac{n^{2}-1}{2n^{2}-1}\right){\left(\mu_{e}-\mu_{g}\right)}^{2}=\frac{2∆f}{hca_{0}^{3}}∆\mu^{2}$$

in which $∆{\tilde{\nu }}_{Stokes}={\tilde{\nu }}_{abs}-{\tilde{\nu }}_{em}$ stands for the Stokes shift, ${\tilde{\nu }}_{abs}$ and ${\tilde{\nu }}_{em}$ are the absorption and emission wavenumbers (cm^−1^) at their intensity maximum, *h* is the Planck’s constant, *c* is the speed of light in vacuum, *a*_0_ is the radius of the Onsager cavity around the fluorophore, *ε* and *n* are the refractive index and the dielectric constant of the solvent, respectively, and $\mu_{e}-\mu_{g}$ (∆μ) is the difference between the dipole moments of the excited and ground states, respectively. Figure 2A displays the fit obtained from the Lippert–Mataga equation. Remarkably, the increase in the solvent polarity causes a lineal rising dependence in the Stokes-shift values, clearly evidencing the influence of a CT phenomenon in the absorption and emission properties of Me_2_CANADC, as previously reported for other electron donor–acceptor functionalized molecules such as substituted tetrahydropyrene derivatives [38]. The Onsager radius was calculated through a Monte-Carlo simulation, and it was estimated to be *a* = 5.16 Å. From the lineal dependency (slope = 8397 cm^−1^, Figure 2A) of the relationship between $∆{\tilde{\nu }}_{Stokes}$ and the solvent orientation polarizability (∆*f*), we calculated the increase in the dipole moment upon electronic excitation and found ∆μ = 10.69 D. The calculated value of the dipole moment at the ground state, $\mu_{g}$, using ACN and THF as environment (continuum model) is 8.05 and 7.67 D, respectively. Using both ∆μ and $\mu_{g}$, values, we extract $\mu_{e}$ value at S_1_: 18.74 and 18.36 D for ACN and THF, respectively. The large value obtained for ∆μ (10.69 D) reflects a strong CT character of the excited molecule. This large charge migration may open interesting photochemistry in the related MOF based on this linker, similar to a photoinduced ligand–metal charge transfer (LMCT) or metal–ligand charge transfer (MLCT) reaction [16,20,21,39].

The fluorescence quantum yields (Φ) of Me_2_CANADC in the different solvents were also measured by using an integrated sphere, and the obtained values are reported in Table 1. There is a negative tendency of the (Φ) values upon increasing the solvent polarity function, as it decreases from 0.40 in DE to 0.10 in MeOH instead. However, this tendency falls out for the DCM solvent, which reaches a maximum of 0.45. This anomalous behavior could be attributed to the effect of the chloride heavy atoms as well as to the fact that DCM cannot present any type of strong interaction with Me_2_CANADC. These observations agree with the involvement of an intramolecular charge transfer (ICT) process at the S_1_ state. Similar ICT processes have been already observed with aromatic molecules containing nitrile and amine groups [38,40,41,42]. Interestingly, the Φ values of Me_2_CANADC in protic polar solvent increase with the number of C atoms in the chain. This could be explained by two facts: (1) as the number of C atoms in the chain increases, the solvent become less polar, thus increasing the Φ value of Me_2_CANADC, and (2) the longer the C chain of the solvent, the higher the viscosity, creating a micro-confinement environment surrounding the Me_2_CANADC, and thus, reducing the non-radiative decay pathways, leading to the observed Φ increase (Table 1). To get more insights on the photophysical properties of Me_2_CANADC and the possible CT processes happening in the excited state, picosecond (ps) time-correlated single photon counting (TCSPC) experiments were carried out.

### 2.2. Time-correlated Single Photon Counting Emission Studies

**Figure 3:**
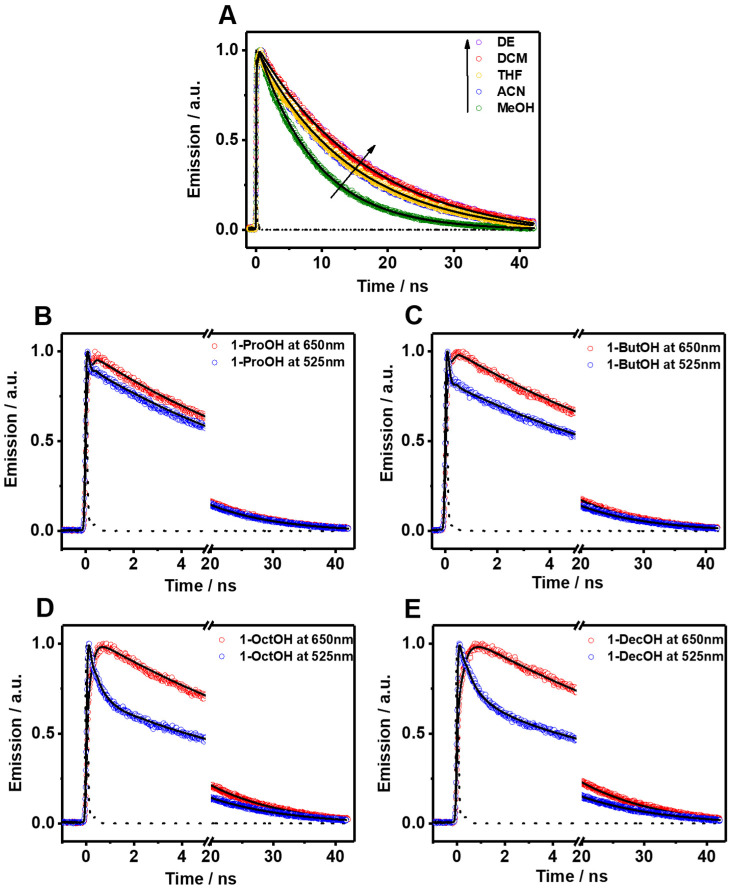
Magic-angle emission decays of Me_2_CANADC in (**A**) DE (purple circles), DCM (red circles), ACN (blue circles), MeOH (green circles), and THF (yellow line) upon 390 nm excitation and observed at 525 nm. Emission decays of Me_2_CANADC in 1-ProOH (**B**), 1-ButOH (**C**), 1-OctOH (**D**), and 1-DeOH (**E**) upon 390 nm excitation and observed at 650 nm (red circles) and 525 nm (blue circles). The solid black lines represent the best-fit using a mono or multiexponential model and the dashed black lines are the instrumental response function (IRF).

The picosecond–nanosecond (ps-ns) fluorescence decays of Me_2_CANADC in the used solvents were obtained by exciting the sample at 390 nm and gating at different wavelengths ranging between 500 and 650 nm. To make the analysis of the result easier, the fluorescence decays were separated into the observed in non-protic (but including methanol) and protic solvents. The fluorescence decays using the first solvents series could be fitted by a global monoexponential function all over the spectral range (Figure 3A and Table 1), whereas the fluorescence decays using the second solvents family exhibit a more complex behavior, and a multiexponential global analysis is required to get accurate fits (Figure 3B–E and Table 2). The monoexponential analyses provided emission lifetime values of 17.6, 17.3, 14.7, 14.2 and 8.9 ns in DE, DCM, THF, ACN, and MeOH, respectively (Table 1). The radiative and non-radiative constants (k_r_ and k_nr_) were calculated and are depicted in Table 1. As we previously mentioned, the increase in the polarity function of the solvent causes a decrease of the fluorescence quantum yield of Me_2_CANADC (with the exception of DCM), and the same effect was also observed for the emission lifetimes. Therefore, as the solvent becomes more polar, the non-radiative constant increases. We observed a very good correlation (R^2^ = 0.97) between the k_nr_ and the solvent orientation polarizability (∆*f*) taking out DCM and ACN solvents, as shown in Figure 2B.

**Figure 4:**
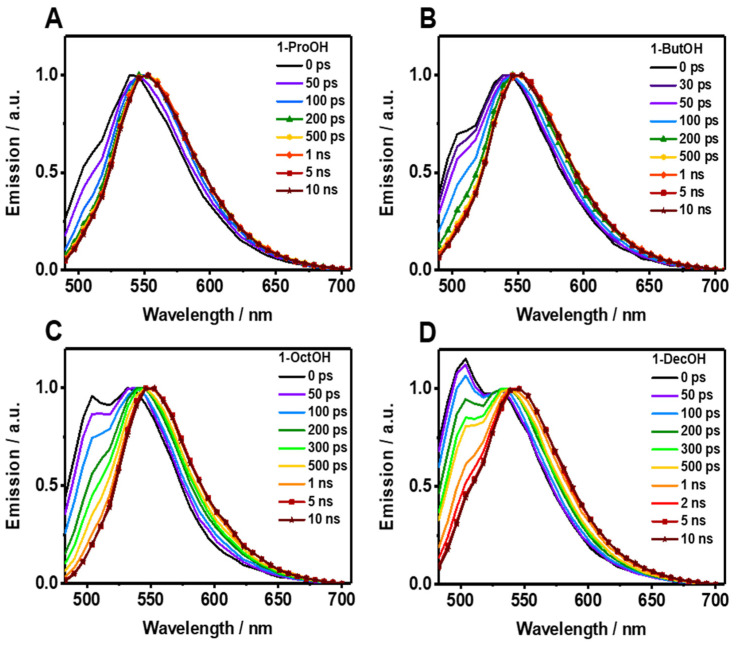
Picosecond time-resolved emission spectra (TRES) of Me_2_CANADC in (**A**) 1-ProOH, (**B**) 1-ButOH, (**C**) 1-OctOH, and (**D**) 1-DeOH at different delays as indicated in the inset. The spectra were normalized on the 550 nm band.

The fluorescence decays of Me_2_CANADC in polar protic solvents are more intriguing, showing a multiexponential behavior in all the cases (Figure 3B–E and Table 2). The decays using 1-ProOH and 1-ButOH are very similar, showing a bi-exponential behavior with time components of τ_1_ = 66 and 85 ps, and τ_2_ = 11.2 and 11.5 ns, respectively. The shortest τ_1_-component vanishes at longer wavelengths; meanwhile, the longest τ_2_-component has its maximum contribution at the reddest part of the spectrum. The fluorescence decays in 1-OctOH and 1-DecOH present an additional component, and the analyses had to be fitted using three components, having time constants of τ_1_ = 160 and 280 ps, τ_2_ = 550 and 955 ps and τ_3_ = 13.1 and 13.8 ns, for 1-OctOH and 1-DecOH, respectively (Table 2). Remarkably, the shortest τ_1_-component is decaying at the bluest part of the spectra but rising at the reddest one, clearly indicating a common channel and the occurrence of a fast photoprocess in the excited state of Me_2_CANADC in these two solvents. The τ_2_-component vanishes at longer wavelengths in similarity to the shortest τ_1_ one previously observed in 1-ProOH and 1-ButOH. This observation could indicate that the τ_2_-component (for 1-OctOH, 1-DecOH) and the τ_1_-component (for 1-OctOH and 1-DecOH) arise from the same excited state species. To get more insights on the spectral position of the emitting species, we recorded the time-resolved emission spectra (TRES) of Me_2_CANADC in the different polar protic solvents (Figure 4). The TRES exhibit a similar shape with an intense band with a maximum at approximately 550 nm visible at all recorded times, and a blue-shifted band having the maximum at approximately 500 nm, which disappears at different delay time depending on the solvent. For instance, this band vanishes in about 60 and 100 ps for 1-ProOH and 1-ButOH, respectively, but it lasts for approximately 500 ps and 1 ns in the case of 1-OctOH and 1-DecOH solvents, respectively (Figure 4). With this knowledge in hand, it is possible to draw a scenario of the excited state photodynamics of Me_2_CANADC in the different polar protic solvents. Upon photoexcitation at 390 nm, Me_2_CANADC is brought to the S_1_ excited state, from where it relaxes by two different pathways: (1) on one side, it deactivates to the S_0_ state from its local excited (LE) state, which is reflected by the short-lived blue emission band at 495 nm; and (2) on the other side, it undergoes a fast-to-ultrafast ICT reaction, and then the species having a CT character relaxes to the S_0_ state emitting light with maximum at 550 nm. This is in agreement with the fact that the emission from the LE state (maximum at approximately 495 nm) is expected to be close to the region of the absorption spectra (position maxima at 436–437 nm, depending on the solvent), which is what we observed herein, with a Stokes shift of just 2680 cm^−1^. In the literature, there are many examples in which the emission from the LE state is different from the core of the molecule, and which is a normal electronic behavior reflecting the effect of the substitution on the solute core. For example, the emission of Prodan (it has the same naphthalene core to that of Me_2_CANADC) from its LE state is a broad band with its maximum of intensity around 475 nm [43]. Similarly, the emission band from the LE state of the very well-known 4,4-dimethylaminobenzonitrile (DMABN) molecule is different from the emission of the benzene core [44]. Therefore, we attribute the shortest component (τ_1_) observed in 1-ProOH and 1-ButOH and the second one (τ_2_) in 1-OctOH and 1-DecOH to the emission lifetime of Me_2_CANADC deactivating from its LE state. The non-contribution of these components to the reddest part of the spectral region, jointly to the vanishing of the blue emission band (500 nm) of the TRES in similar times (60 ps, 100 ps, 500 ps and 1 ns for 1-ProOH, 1-ButOH, 1-OctOH, and 1-DecOH, respectively), clearly evidence the hit of our assignment. Therefore, the longest component (τ_3_) can be attributed to the emission lifetime of the species having a CT character, as its maximum contribution is at the reddest part of the spectrum. The emission lifetime of these CT species increases with the viscosity of the solvent, as previously observed for the quantum yield values, supporting our previous explanation involving a micro-confinement environment in which a higher viscosity of the solvent surrounding the Me_2_CANADC reduces the non-radiative constant. Finally, the shortest time component (τ_1_) recorded in 1-OctOH and 1-DecOH, which is decaying at the higher energies but rising at the lower ones, is the signature of an ICT process. This event is less favored (become slower) with the viscosity of the solvent, and it takes place in times shorter than our system resolution (non-detectable) for solvents with lower viscosity than 1-OctOH. The slowing down of the ICT reaction at higher viscosity of the solvent can be explained by the micro-confinement effect of the environment. However, we cannot completely rule out the possibility of a twisted ICT reaction involving the amino group. For instance, amino-substituted molecules such as 4,4-dimethylaminobenzonitrile (DMABN) have shown dual fluorescence from an LE state and a CT state generated by a twisted ICT, where indeed, the internal twisting of the dimethylamino group along with a CT from the amino nitrogen to the in-plane π orbital of the cyano group is responsible for the red-shifted emission band [44,45]. The deceleration of the ICT reaction for Me_2_CANADC in 1-OctOH and 1-DecOH solvent could be also the reason of the long-lived emission observed from the LE state. 

**Scheme 2:**
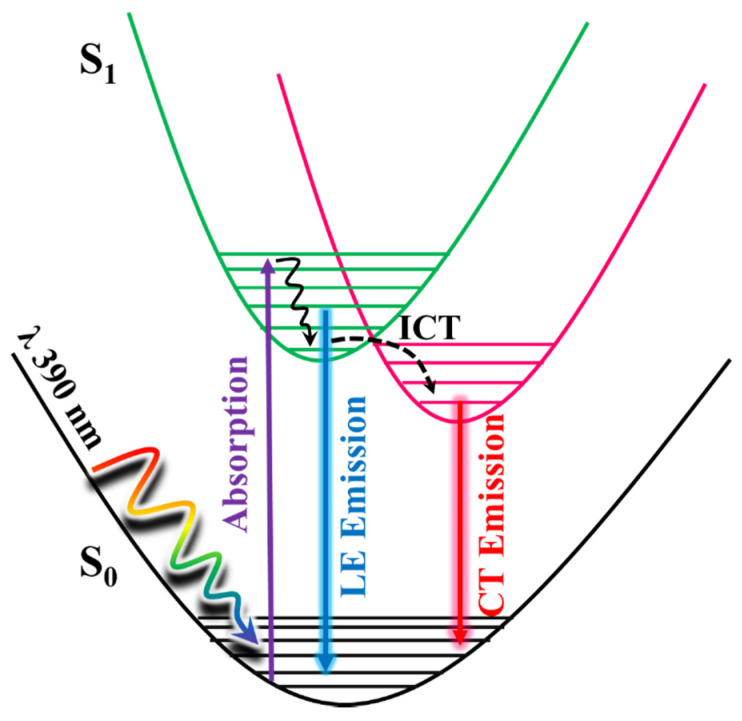
Schematic representation of the photodynamics processes happening in the photoexcited Me_2_CANADC molecule. The scheme displays the ultrafast intramolecular charge transfer (ICT) reaction alongside the relaxation from the local excited (LE) and the ICT states.

Based on all the above, the photodynamics happening upon photoexcitation of Me_2_CANADC is illustrated in Scheme 2. For the non-protic solvents and methanol, after photoexcitation, an ultrafast ICT takes place, inhibiting the relaxation of Me_2_CANADC from its LE state in the picosecond–nanosecond regime and leading to the formation of CT species. To get more information about the ultrafast ICT reaction happening in non-protic solvents and methanol, femtosecond (fs) transient absorption experiments were performed.

### 2.3. Femtosecond Time-resolved Transient Absorption Studies

**Figure 5:**
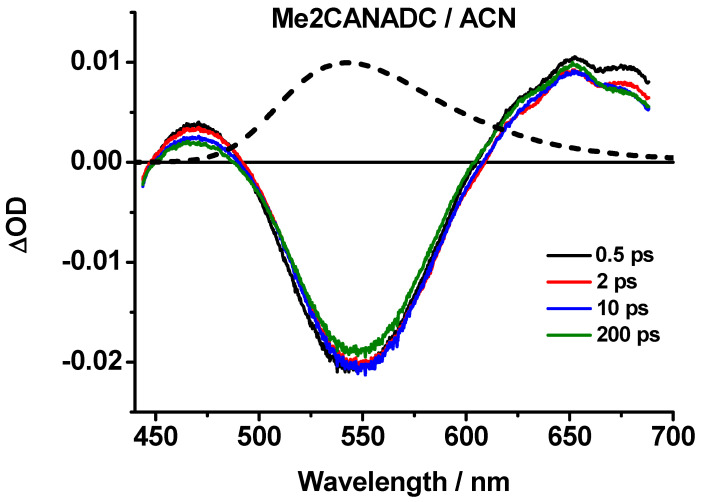
Femtosecond transient absorption spectra (TAS) of Me_2_CANADC in ACN solvent at different time delays upon excitation at 420 nm. The dashed spectrum corresponds to the steady-state emission one showing the good overlap with the negative TAS band.

The ultrafast photoevents in Me_2_CANADC in DE, THF, DCM, ACN, and MeOH were explored by means of fs-transient absorption spectroscopy, pumping the sample at 420 nm and gating the related transient spectra and dynamics in a large spectral range (450–700 nm). The obtained transient absorption spectra, shown in Figure 5 and Figure S1 (Supplementary Materials), can be divided into sub-picosecond and picosecond time domains. In the sub-picosecond interval (0.5 ps), we observed a positive band with a maximum intensity at 525–550 nm depending on the solvent, whereas the spectra in the ps time domain are all characterized by an intense negative band centered at 525–550 nm and two positive bands at approximately 475 nm and 650 nm (Figure 5 and Figure S1). The bands remain visible even after 200 ps. Additionally, the negative band well overlaps with the emission bands of Me_2_CANADC collected in the steady-state experiments; thus, this signal can be undoubtedly attributed to its stimulated emission. The emission from the CT state is much stronger than that of the LE, reflecting the ultrafast ICT process (should be shorter than 0.5 ps), and therefore, the steady-state and the stimulated emission spectra gated after 0.5 ps time should not be very different in shape, as shown in Figure 5. In addition to that, the recorded negative band is certainly a combination of stimulated emission (which is negative) and the transient absorption (which is positive) of the formed CT structure. Following the observation, it is clear that the stimulated emission intensity is much stronger than the absorption one.

**Figure 6:**
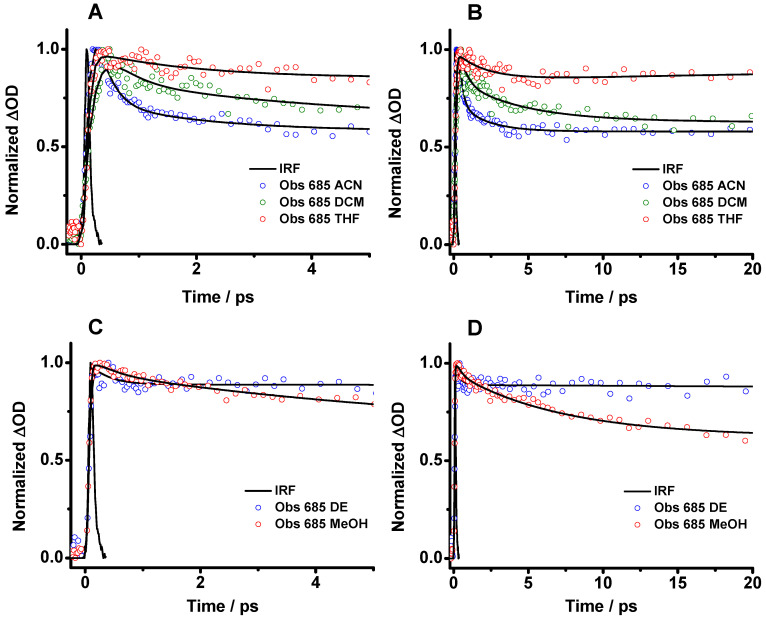
Femtosecond transient absorption decays of Me_2_CANADC in (**A**,**B**) ACN, DCM and THF and (**C**,**D**) DE and MeOH at different time windows. The solid lines are from the best multiexponential fits (see Table 3). The decays were recorded at 685 nm, and the samples were excited at 420 nm.

As the strong negative band interferes with the whole transient signal and prevents a detailed study of the photobehavior of Me_2_CANADC, the transient decays were analyzed at 685 nm in order to reduce as much as possible the signal from the stimulated emission (Figure 6 and Figure S2). The analysis of the transient decays of Me_2_CANADC in all the used solvents reveals a multiexponential behavior with different ultrafast time components and a long tail offset that does not decay within 200 ps (Figure 6 and Figure S2 and Table 3). The long offset decay (not presented in Table 3) coincides with the contribution of the long-lived emission (8–17 ns) of the structure having a CT character, as previously described in the TCSPC part. In THF, DCM, ACN and MeOH, the decays present three-time components of τ_1_ = 70–195 fs, τ_2_ = 0.37–2.06 ps, and τ_3_ = 1.82–23 ps (Table 3). While τ_1_ has a negative contribution, indicating that is a rising component, τ_2_ and τ_3_ are decaying (positive contribution). Therefore, the rising τ_1_ component can be assigned to the ultrafast ICT process from the LE state. The value of this component does not have a clear correlation with the macroscopic properties of the solvents, but for DCM, ACN, and MeOH, it decreases accordingly with the polarity function value. In the case of DE and THF, the interaction of the O groups of the solvents with the NH_2_ of the Me_2_CANADC molecule may modify somehow the ICT reaction, making it faster. The τ_2_ and τ_3_ components could be the consequence of the intramolecular vibrational energy redistribution (IVR) and the solvent relaxation phenomena, as previously observed for other organic molecules [46,47,48,49]. However, we cannot discard that the transient absorption transition from the LE state is hidden in any of these components. Based on these results, we demonstrate that in the case of the aprotic solvents and methanol, the relaxation of excited Me_2_CANADC from its LE state to the S_0_ cannot compete with the ultrafast ICT reaction, and upon photoexcitation, the species having CT character are promptly formed. Once the ultrafast photodynamics of Me_2_CANADC in the different solvents were deciphered, the possible formation of a long-lived charge separated state in the photoexcited molecule has been explored by using a flash photolysis technique.

### 2.4. Flash Photolysis Transient Absorption Studies

**Figure 7:**
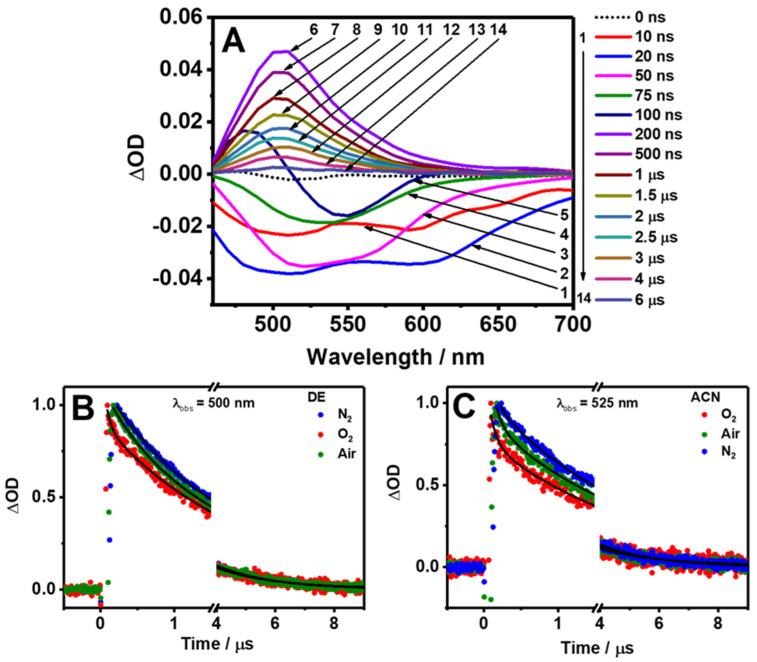
(**A**) Time-resolved transient absorption spectra of Me_2_CANADC in DE at different delay times upon excitation at 420 nm. (**B**,**C**) Transient decays of Me_2_CANADC in DE and ACN under different atmospheric conditions: air (green), oxygen (red), and nitrogen (blue). The observation wavelengths are indicated in the figure. The solid black lines are obtained from the best fit using a mono or multiexponential functions.

The slow relaxation dynamics of Me_2_CANADC have been explored by performing flash photolysis TA experiments (ns-ms regime) pumping the sample at 420 nm and probing it at 525 nm for ACN (Figure 7C), THF, and MeOH (Figure S3), and at 500 nm for DE (Figure 7B) and DCM (Figure S3). The collected TA spectra of Me_2_CANADC in DE clearly show two different behaviors at short and long delay time domains (Figure 7A). At early delays (up to 100 ns), the spectra are dominated by a negative signal, which can be attributed to the fluorescence emission of Me_2_CANADC. Interestingly, at the initial time delays (from 10 to 20 ns), two negative bands centered at 500 and 600 nm with a growing intensity were detected, which later turned into a single negative band whose intensity decreases up to a delay time of 100 ns. After this time delay, the raise of a positive absorption band centered at 500 nm is observed. This positive band became the dominating signal of the spectra having its maximum intensity at a delay time of 200 ns and decreasing its intensity from then until its complete disappearance at times longer than 6 µs. 

To get more knowledge on the slow relaxation mechanism of Me_2_CANADC, the transient decays under normal air conditions and in saturated O_2_ and N_2_ atmospheres (bubbling the solution with the gases) were recorded. Firstly, the negative part of the signal (early time delays, <200 ns) was investigated under air and oxygen atmospheres by probing the sample at 650 nm in order to minimize any possible contribution from the positive signal of the spectra (Figure S4). The negative decays can be fitted to a monoexponential function in all the used solvents and in both atmospheres (air and O_2_); however, the time component values diminishes from the decays obtained in air (τ = 14–24 ns, Table S1) to the ones obtained in a saturated O_2_ environment (τ = 10–16 ns, Table S1). Notably, this time constant obtained from the negative part of the spectra nicely matches with the lifetime obtained from the TCSPC experiments (vide supra), and thus it is attributed to the stimulated fluorescence lifetime of the Me_2_CANADC having a CT character. This assignment is further supported by the quenching of this time component when the Me_2_CANADC solutions are in a saturated O_2_ atmosphere (Figure S4). Comparable quenching of the CT state has been previously reported for other donor–acceptor molecules in the presence of oxygen [50]. 

Then, we investigated the transient decays associated to the positive absorption band collected at longer delay times (200 ns–6 µs) under different atmospheric (air, N_2_, and O_2_) conditions (Figure 7B,C and Figure S3). The long-lived decays present a bi-exponential behavior in the different solvents and atmospheric conditions. However, under saturated N_2_ atmosphere, the transient decays in DE, DCM, and ACN only required a monoexponential analysis to be well fitted. The shortest τ_1_ component is strongly influenced by the presence of oxygen. For instance, in THF, the value of τ_1_ decreases from 501 ns in the absence of oxygen (N_2_ atmosphere) to 236 and 29 ns under air and saturated O_2_ atmospheres, respectively. A similar tendency is observed for the rest of the solvents in which the τ_1_ value in air is longer (564–104 ns, depending on the solvent) than the one recorded under O_2_ (172–5 ns, Table 4). The quenching of this component with the presence of oxygen may indicate the involvement of a triplet (T_1_) state, so this short component could be attributed to a transient absorption transition from a non-emissive T_1_ excited state. The existence of a T_1_ state has been previously reported for the non-functionalized (NDC) and the monofunctionalized (NADC) molecules, having both the same naphthalene dicarboxylate core structure [20]. It has been also described for DMABN, whose central core is a benzene ring instead of naphthalene, but which is double functionalized with an amino and carbonitrile groups, similar to Me_2_CANADC [51,52,53,54]. On the other hand, the longest τ_2_ component is unaffected by the presence of oxygen and exhibits a similar value of approximately 2 µs in all the studied solvents and environments (Table 4). It is very well known that the photoexcitation of a push–pull (electron donor–acceptor) archetype system can generate long-lived charge separated (CS) states [55,56]. Based on that, it is very plausible that the 2 µs component arises from a long-lived CS state that is produced after the photoexcitation of Me_2_CANADC and as a consequence of the ultrafast ICT. The formation of this long-lived CS state is of vital importance for future optoelectronic applications as well as for solar energy conversion and/or photocatalysis processes. In principle, it could be possible to separate the spectra of the T_1_ state from the CS one; however, to do so, we must record the transient absorption spectra of the sample, which is saturated with oxygen, to speed up the deactivation from the T_1_ state upon collision with the molecular oxygen, and record only that of the CS state. Unfortunately, under these conditions, the sample photodegrades during the time of the experiment (2 h). However, the sample was stable enough to get single wavelength decays (10–15 min). We also tried to combine multiple single decays to construct the spectra, but the results were not satisfactory (the signal-to-noise ratio was not good). 

Summarizing, after photoexcitation, the long-lived photodynamics of Me_2_CANADC is characterized by a negative fast signal (in the order of 15 ns) corresponding to the stimulated fluorescence lifetime of the CT character species, and two positive transient absorption transitions from a non-emissive T_1_ state (from few ns to hundreds of ns depending on the conditions) and from a CS state (approximately 2 µs), respectively. 

## 3. Experimental part

### 3.1. Materials

Anhydrous dichloromethane (≥99.8%), diethyl ether (≥99.9%), acetonitrile (99.8%), methanol (≥99.9%), tetrahydrofuran (≥99.9%), 1-propanol (99%), 1-butanol (99.5%), 1-octanol (99%), and 1-decanol (99%) were purchased from Sigma-Aldrich (Madrid, Spain) and used as received. Oxygen and nitrogen (premier X-50S) for the time-resolved experiments were purchased from Carburos Metalicos (Aranjuez, Spain). 

### 3.2. Synthesis of Dimethyl 4-amino-8-cyanonaphthalene-2,6-dicarboxylate (Me_2_CANADC) 

Dimethyl 4-amino-8-cyanonaphthalene-2,6-dicarboxylate (Me_2_CANADC) was prepared in three successive steps from available dimethyl 4-bromonaphthalene-2,6-dicarboxylate (Scheme S1, Supplementary Materials) [57]. At first, a substitution from bromo to cyano by zinc cyanide in the presence of a palladium catalyst, which provided a better yield and easier experimental procedure than previously described. In the second step, in the dimethyl 4-cyanonaphthalene-2,6-dicarboxylate, a nitro group was selectively introduced to position 8 in the naphthalene ring at low temperature using nitric acid in sulfuric acid media. Finally, we performed the reduction of a nitro to amino group by hydrogen catalyzed by Pd/C in an acetic acid/dioxane mixture and purified the obtained product by recrystallization.

### 3.3. Methods

#### 3.3.1. Materials Characterization Techniques 

The naphthalene dicarboxylates were characterized by ^1^H and ^13^C nuclear magnetic resonance, Fourier Transform Infrared (FTIR) and High-Resolution Mass (HR-MS) spectroscopies (see Supplementary Materials for more information).

#### 3.3.2. Steady-state and Time-resolved Characterization Techniques

Me_2_CANADC was dissolved in the selected organic solvents and the solutions were adjusted to have an optical density of 0.3 at their maximum absorption intensity in order to avoid undesired effects (inner filtering, aggregates, etc.). The steady-state absorption and fluorescence measurements were carried out using JASCO V-670 and FluoroMax-4 (Horiba Jobin-Yvon, Paris, France) spectrophotometers, respectively. A Quanta-φ integrating sphere (Horiba) was connected to the FluoroMax-4 emission spectrophotometer to measure the quantum yields. 

The picosecond-nanosecond (ps-ns) emission decays and time-resolved emission spectra were collected by means of a time-correlated single photon counting system (TCSPC). The samples were excited by a 40-ps pulsed laser centered at 390 nm (4 mW, 40 MHz repetition rate). The emission signal was collected at the magic angle (54.7°), and the instrumental response function (IRF) recorded using a LUDOX suspension was approximately 75 ps. The decays were deconvoluted and fitted with mono- and multiexponential models using the FLUOFIT software that allows single and global analyses. The quality of the fit was estimated through the value of the χ^2^, which was kept below 1.2, and the residual contribution. 

The femtosecond transient absorption experiments were done using a chirped pulse amplification setup. In brief, it comprises a Ti:Sapphire oscillator (TISSA 50, CDP Systems) pumped by a 5 W diode laser (Verdi 5, Coherent) that seeds (800 nm, 30 fs, 450 mW at 86 MHz) a regenerative amplifier (Legend-USP, Coherent). The amplified fundamental beam (50 fs, 1 W at 1 kHz) pumps an optical parametric amplifier for wavelength conversion (CDP Systems). The second harmonic (400 nm) of the fundamental beam or the fourth harmonic (350 nm) of the OPA output were used as a pump. The pump pulse intensity was kept below approximately 200 µW. The instrument response function (IRF) was measured in terms of ∆OD for DCM following excitation at 350 and 400 nm to give 120 and 100 fs, respectively. The samples were placed in a 1-mm quartz spinning cell to avoid re-excitation and fast photodegradation by consecutive laser pulses. 

Nanosecond flash photolysis decays and associated spectra were obtained using an LKS.60 laser flash photolysis spectrometer (Applied Photophysics, Leatherhead, United Kingdom) and a Vibrant (HE) 355 II laser (Opotek) as pump. The samples were excited using the third harmonic (355 nm) output and the signal from the optical parameter oscillator (OPO, pumped by a Q-switched Nd:YAG laser, Brilliant, Quantel) at 420 nm. The probe was a 150 W Xenon arc lamp, and the resulting signal transmitted through the sample was dispersed by a monochromator and detected by a visible photomultiplier (Applied Photophysics, R928) connected to a digital oscilloscope (Agilent, Infiniium DS08064A, 600 MHz, 4 GSa s1). The IRF was about 8 ns. The samples were studied under air, oxygen, and nitrogen atmospheres, bubbling the gases into the solutions for 5 min prior to perform the measurements. All the measurements were done at 298 K using a 1 cm quartz cuvette.

## 4. Conclusions

In this work, we have reported on the rich photodynamics of a potential linker for future MOFs engineer, Me_2_CANADC. This molecule, having a naphthalene central core, is doubled functionalized with an electron donor and acceptor moieties, leading to a push–pull archetype system with very promising optical properties. We have demonstrated that the optical absorption and emission behavior of Me_2_CANADC is strongly influenced by the surrounding environment, owing to the excited-state CT character of this molecule. Indeed, from the TCSPC experiments, we have unveiled that the steady-state emission is originated by these CT character species, emitting with a lifetime of about 8–15 ns; however it is also possible to detect the blue-shifted and short-lived emission from the LE state of the molecule in polar protic solvents. From the femtosecond transient absorption experiments, the ultrafast ICT reaction after photoexcitation has been determined to occur in just tens of femtoseconds. The slow photodynamics of a photoexcited Me_2_CANADC molecule was also explored. At short delay times (few ns), a negative signal resulting from the stimulated emission of Me_2_CANADC having a CT character was detected. At longer delay times, a positive transient absorption signal associated to two decay components was observed. The first component, in the range of hundreds of ns, was strongly influenced by the presence of oxygen, which quenches its value, being this a clear indication of the involvement of a non-emissive T_1_ state, similarly to its mother and sister non-functionalized and mono-functionalized molecules (NDC and NADC respectively). Finally, the longest microsecond component was attributed to the formation of a long-lived charge separated state, having this a special importance for the future role of this linker in the fabrication of optoelectronically active MOFs. Our results demonstrate the importance of the functionalization of organic linkers to tune their optical and ultrafast photophysical properties, opening an exciting pathway for the development of functional linkers for the further fabrication of smarter and more efficient MOFs. 

## Figures and Tables

**Table 1:** Solvent properties and spectroscopic data of Me_2_CANADC.

Solvent	Number	∆*f*	η (cP)	λ_abs_ (nm)	λ_em_ (nm)	Stokes Shift (cm^−1^)	Φ	τ (ns)	k_r_^b^ (10^7^ s^−1^)	k_nr_^b^ (10^7^ s^−1^)
**DE**	1	0.162	0.23	425	512	3998	0.40	17.6	2.27	3.41
**THF**	2	0.220	0.48	431	534	4475	0.28	14.7	1.90	4.90
**DCM**	3	0.230	0.43	408	506	4747	0.45	17.3	2.54	3.24
**ACN**	4	0.330	0.35	419	542	5416	0.16	14.2	1.06	5.99
**MeOH**	5	0.333	0.58	430	559	5367	0.10	8.9	1.12	10.11
**1-ProOH**	6	0.296	2.22	436	557	4982	0.14	11.2 ^a^	1.25	7.68
**1-ButOH**	7	0.286	2.90	436	557	4982	0.15	11.5 ^a^	1.30	7.39
**1-OctOH**	8	0.241	8.46	436	553	4853	0.20	13.1 ^a^	1.53	6.11
**1-DecOH**	9	0.217	13.19	437	551	4734	0.25	13.8 ^a^	1.81	5.43

Values of the solvent orientation polarizability (∆*f*) and viscosity η (at 20 °C), wavelengths of the intensity maxima of absorption and emission bands, Stokes-shift value, fluorescence quantum yields recorded with a 400 nm excitation wavelength, fluorescence lifetimes upon excitation at 390 nm and observation at 425 nm, and the calculated radiative and non-radiative rate constants of Me_2_CANADC in each solvent. ^a^ We give only the longest fluorescent lifetime. More details are given in Table 2. ^b^ These values were calculated using the longest fluorescent lifetime.

**Table 2:** Time constant τi, normalized (to 100), pre-exponential factor Ai and fractional contributions (Ci = τiAi) extracted from the fit of the emission decays of Me_2_CANADC in 1-ProOH, 1-ButOH, 1-OctOH, and 1-DeOH upon excitation at 390 nm. The estimated error is about 15%.

Solvent	λ_obs_/nm	τ_1_/ps	A_1_ (%)	C_1_ (%)	τ_2_/ps	A_2_ (%)	C_2_ (%)	τ_3_/ns	A_3_ (%)	C_3_ (%)
**1-ProOH**	500	66	75	2	-	-	-	11.2	25	98
	525	66	49	1	-	-	-	11.2	51	99
	550	66	28	<1	-	-	-	11.2	72	>99
	575	66	13	<1	-	-	-	11.2	87	>99
	600	-	-	-	-	-	-	11.2	100	100
	650	-	-	-	-	-	-	11.2	100	100
**1-ButOH**	500	85	71	2	-	-	-	11.5	29	98
	525	85	52	1	-	-	-	11.5	48	99
	550	85	27	<1	-	-	-	11.5	73	>99
	575	-	-	-	-	-	-	11.5	100	100
	600	-	-	-	-	-	-	11.5	100	100
	650	-	-	-	-	-	-	11.5	100	100
**1-OctOH**	500	160	61	3	550	14	2	13.1	25	95
	525	160	33	1	550	17	1	13.1	50	98
	550	160	−100	−100	550	14	1	13.1	86	99
	575	160	−100	−100	-	-	-	13.1	100	100
	600	160	−100	−100	-	-	-	13.1	100	100
	650	160	−100	−100	-	-	-	13.1	100	100
**1-DecOH**	500	280	60	4	955	12	3	13.8	28	93
	525	280	31	1	955	15	2	13.8	54	97
	550	280	−100	−100	955	9	1	13.8	91	99
	575	280	−100	−100	-	-	-	13.8	100	100
	600	280	−100	−100	-	-	-	13.8	100	100
	650	280	−100	−100	-	-	-	13.8	100	100

**Table 3:** Time constants (τ_i_) and normalized (to 100) pre-exponential factors (A_i_) extracted from the fits of the femtosecond (fs)-transient absorption decays of Me_2_CANADC excited at 420 nm and probed at 685 nm. A negative value of Ai indicates a rising component in the signal. The estimated error is about 15%.

Solvent	τ_1_ (fs)	A_1_ (%)	τ_2_ (ps)	A_2_ (%)	τ_3_ (ps)	A_3_ (%)
DE	50	−100	0.33	100	-	-
THF	100	−93	2.06	100	23	−7
DCM	195	−00	0.37	68	4.1	32
ACN	95	−100	0.22	36	1.8	64
MeOH	70	−100	0.54	30	9.5	70

**Table 4:** Time constants (τ_i_), pre-exponential factors (A_i_) extracted from the transient absorption decays fits of Me_2_CANADC in ACN, DCM, DE, MeOH, and THF observed at 500–525 nm upon excitation at 420 nm. The estimated error is around 15%.

Solvent	N_2_				Air				O_2_			
	τ_1_ (ns)	A_1_ (%)	τ_2_ (µs)	A_2_ (%)	τ_1_ (ns)	A_1_ (%)	τ_2_ (µs)	A_2_ (%)	τ_1_ (ns)	A_1_ (%)	τ_2_ (µs)	A_2_ (%)
DE	-	-	1.8	100	444	15	2.0	85	64	33	1.9	67
THF	501	11	2.0	89	236	18	2.0	82	29	74	1.9	26
DCM	-	-	1.8	100	564	12	1.9	88	172	15	1.9	85
ACN	-	-	1.9	100	104	42	2.0	58	90	39	2.0	61
MeOH	28	93	2.0	7	111	38	2.0	62	5	99	2.0	1

## Supplementary Materials

Supplementary Materials can be found at https://www.mdpi.com/1422-0067/21/12/4366/s1.
