# Supplementary material for: Femto- to Millisecond Time-Resolved Photodynamics of a Double-Functionalized Push–Pull Organic Linker: Potential Candidate for Optoelectronically Active MOFs
Source: Int J Mol Sci. 2020 Jun 19;21(12):4366. doi: 10.3390/ijms21124366 (PMC7352538; doi:10.3390/ijms21124366)
Supplement: Supplementary file 1 [file ijms-21-04366-s001.pdf]

# SUPPLEMENTARY MATERIALS FOR

*Article*

# Steady-State and Time-Resolved Photodynamics Characterization of a Double-Functionalized Push-Pull Organic Linker: Potential Candidate for Optoelectronically Active MOFs

## Materials

Solvents were dried by standard methods or by elution using a PureSolv Innovative Technology drying system. All reagents and solvents were commercially available and used without further purification unless otherwise indicated and dimethyl 4-bromonaphtahlene-2,6-dicarboxylate was synthetized as described.[i]

## Characterization Methods

$^{1}$H and $^{13}$C nuclear magnetic resonance spectra were recorded with a VARIAN INOVA 300. The HR-MS analysis was carried out by using an Agilent 1200 Series LC system coupled to a 6520 quadrupole-time of flight (QTOF) mass spectrometer. The ionization source was an ESI interface working in the positive-ion mode and Infrared (FTIR) spectra were recorded on a FT-PerkinElmer Spectrum One.

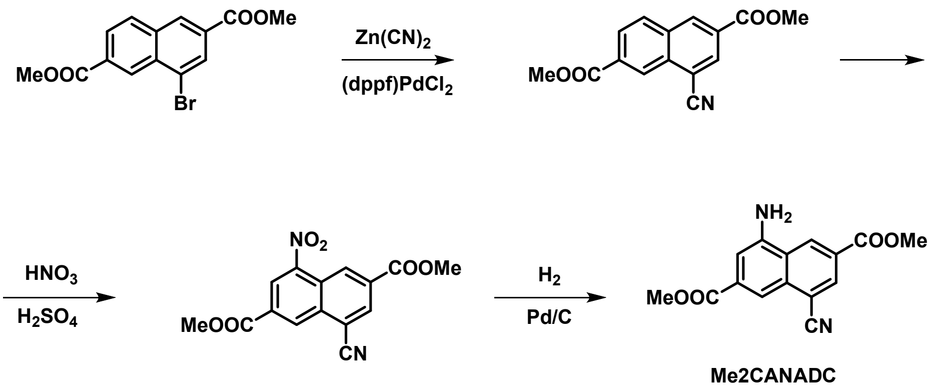


**Scheme S1.** Synthesis of dimethyl 4-amino-8-cyanonaphthalene-2,6-dicarboxylate (Me2CANADC).

## Synthetic Procedures and Characterization Data

Dimethyl 4-cyanonaphthalene-2,6-dicarboxylate[1]

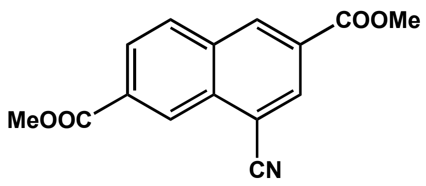


A solution of dimethyl 4-bromo-naphthalene-2,6-dicarboxylate (3 g, 9,28 mmol) and PMHS (200 mg) in dimethylacetamide (10 mL) was heated at 120ºC. (dppf)$PdCl_2$ (135 mg, 0.19 mmol, 2%) and then zinc cyanide (600 mg, 5.1 mmol. 55%) were added. The reaction was monitorized by TLC. After the reaction was completed, solution was diluted with ethyl acetate (200 mL) and filtered for removing metal salts. The organic layers were exahustively washed with water, and brine, dried under sodium sulfate and concentrate under reduced pressure. The solid was recristallized from acetic acid/methanol (1:1) obtaining 1.95 g (78%)

of pure dicarboxylate that present the same $^{1}$H-NMR and FTIR of that described before by non catalyzed substitution using CuCN in DMF.

$^{1}$H NMR (300 MHz, Chloroform-d) δ 8.96 (s, 1H), 8.81 (s, 1H), 8.54 (d, J = 1.6 Hz, 1H), 8.28 (dd, J = 8.6, 1.6 Hz, 1H), 8.10 (d, J = 8.6 Hz, 1H), 4.03 (s, 6H). FT-IR (cm$^{-1}$) υ 2960 (CH), 2228 (CN), 1725 (C=O), 1437, 1280 (C-O), 1206, 1101.

Dimethyl 4-cyano-8-nitronaphthalene-2,6-dicarboxylate

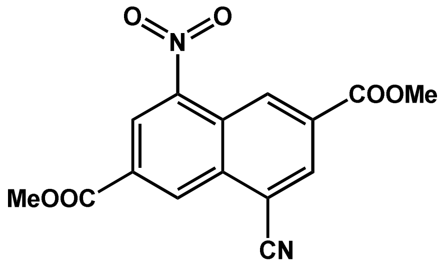


Dimethyl 4-cyanonaphthalene-2,6-dicarboxylate (1.2 g, 4.46 mmol) was disolved in sulfuric acid (25 mL) by slowly addition at -5ºC. Then a solution of 65% nitric acid (0.74 mL, 10.7 mmol, 2.4 eq.) in sulfuric acid (5 mL) was dropwise added. The reaction was monitorized by TLC (Heptane/Ethyl acetate; 2:1). When the reaction was completed, the solution was poured on ice-water with efficient stirring. The precipitate was filtered, washed with water, dried and recristallized from acetic acid/methanol to obtain 1.19 g ( 85%) of pure dicarboxylate.

$^{1}$H NMR (300 MHz, Chloroform-d) δ 9.46 (s, 1H), 9.24 (s, 1H), 8.93 (s, 1H), 8.71 (s, H), 4.09 (s, 3H), 4.07 (s, 3H). $^{13}$C NMR (75 MHz, Chloroform-d) δ164.34, 164.06, 148.15, 134.34, 134.22, 132.69, 132.04, 131.26, 130.43, 126.44, 125.52, 115.86, 113.42, 53.59. FT-IR (cm$^{-1}$) υ 2961 (CH), 2233 (CN), 1725,(C=O), 1540 (NO2), 1437 (NO2), 1295, 1213 (C-O), 1108. HR-MS analysis: Calculated for $C_{15}H_{10}N_2O_6$ = 314.05389, founded = 314.05246.

Dimethyl 4-amino-8-cyanonaphthalene-2,6-dicarboxylate

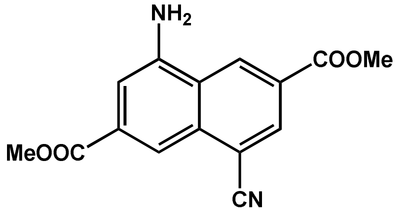


To a solution of dimethyl 4-cyano-8-nitronaphthalene-2,6-dicarboxylate (1 g, 3.18 mmol), a mixture of dioxane/acetic acid (20 mL/50 mL) was carefully added 10% Pd/C (102 mg, 3% Pd) dispersed in acetic acid (1mL) and pressurized with hydrogen (1.1 atm). After efficient stirring for 4 hours at room temperature, the reaction mixture was filtered through a short pad of celite and concentrated to dryness to obtain 860 mg, (>95%) of dimethyl 4-amino-8-cyanonaphthalene-2,6-dicarboxylate as yellow-orange fluorescent solid.

$^{1}$H NMR (300 MHz, DMSO-d6) δ 9.14 (s, 1H), 8.46 (s, 1H), 7.82 (s, 1H), 7.40 (s, 1H), 6.88 (s, $N\underline{H}_2$, 2H), 3.94 (s, 3H), 3.92 (s, 3H). $^{13}$C NMR (75 MHz, DMSO-d6) δ 166.09, 164.92, 148.79, 134.02, 132.62, 132.62, 130.70, 125.73, 122.51, 117.16, 111.44, 110.60, 108.71, 52.73, 52.66. FT-IR (cm$^{-1}$) υ 3472, 3378 (NH), 2958 (CH), 2224 (CN), 1725 (C=O), 1438, 1303, 1248 (C-O), 1101. HR-MS analysis: Calculated for $C_{15}H_{12}N_2O_4$ = 284.07971, founded = 284.08070.

**Characterization data of naphthalene-2,6-dicarboxilates**

Dimethyl 4-cyanonaphthalene-2,6-dicarboxylate

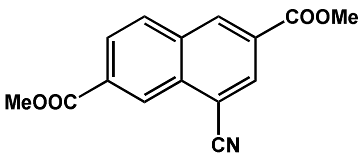

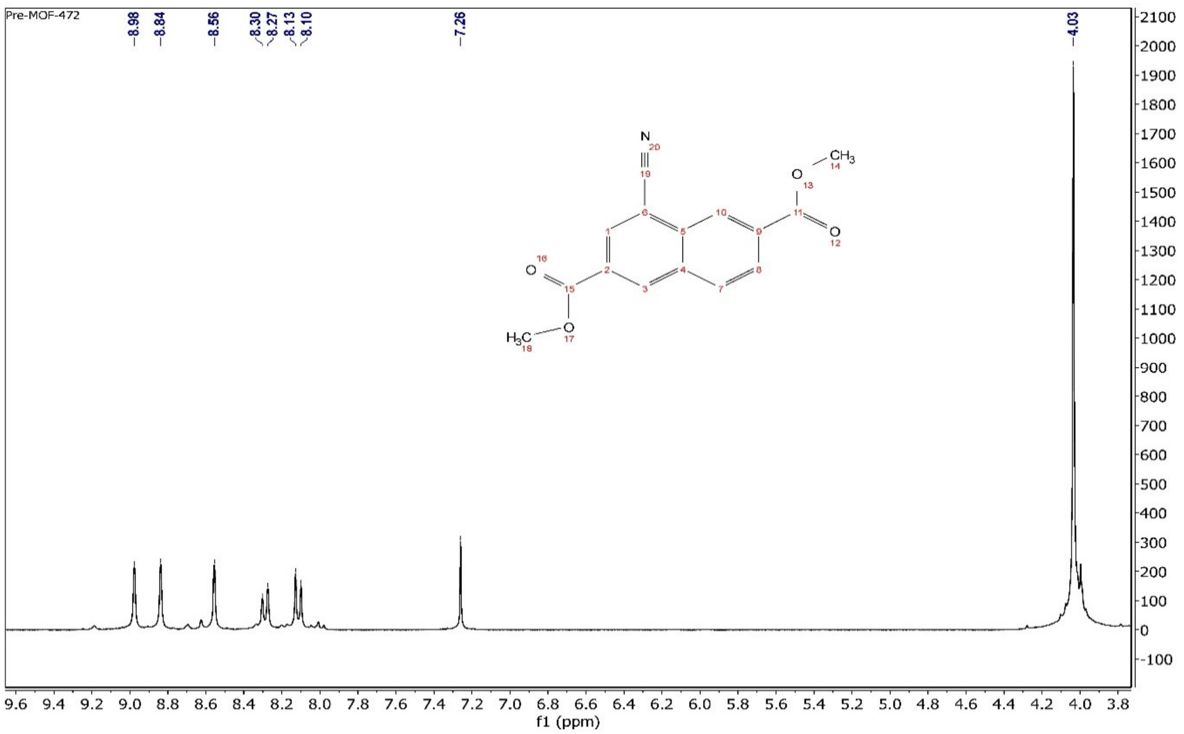
Pre-MOF-472
8.98
8.84
8.56
8.30
8.27
8.13
8.10
7.26
4.03
f1 (ppm)

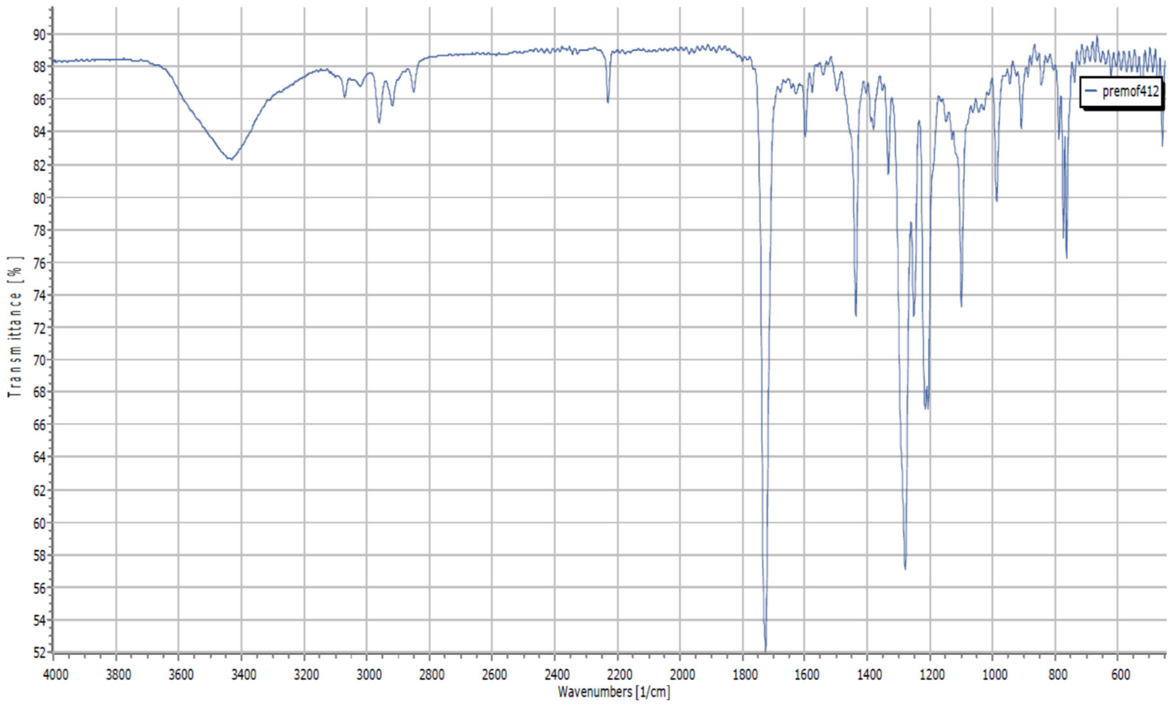
premof412
Transmittance [%]
Wavenumbers [1/cm]

Dimethyl 4-cyano-8-nitronaphthalene-2,6-dicarboxylate

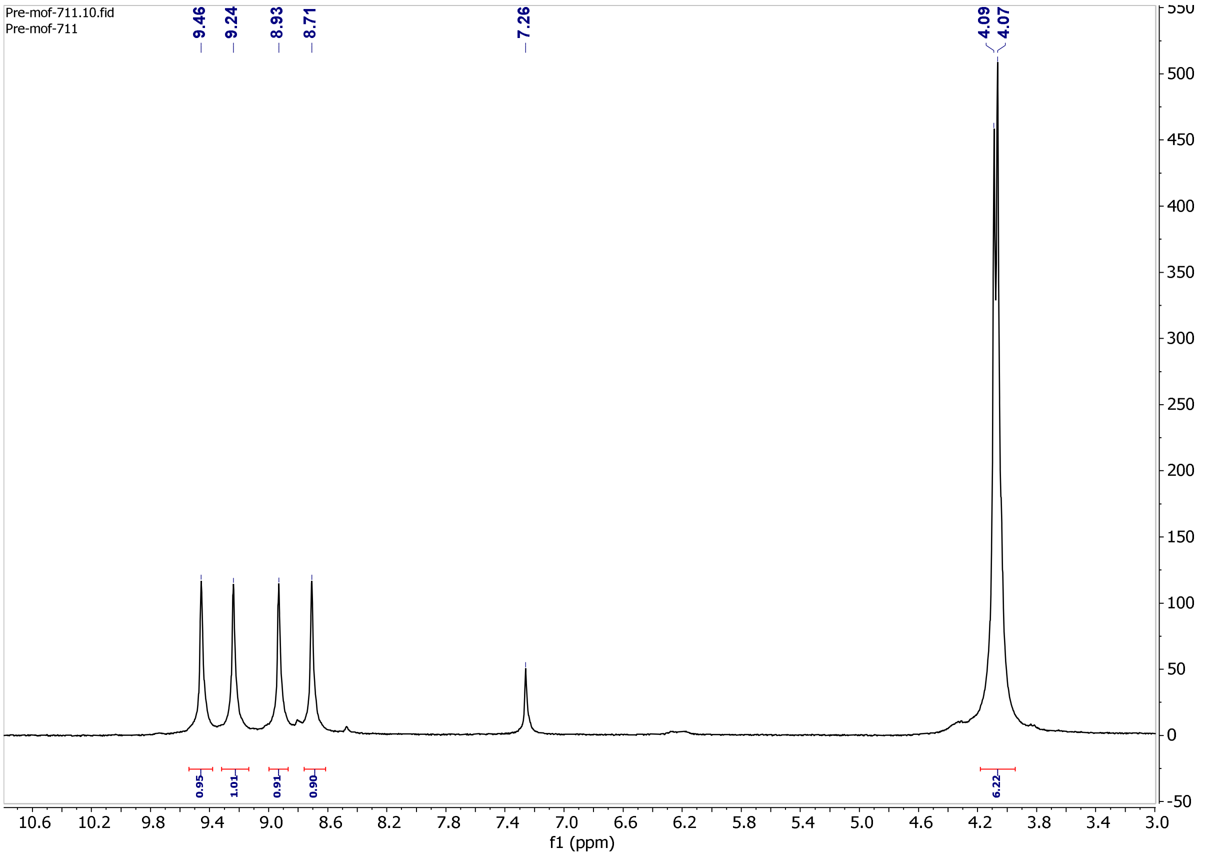


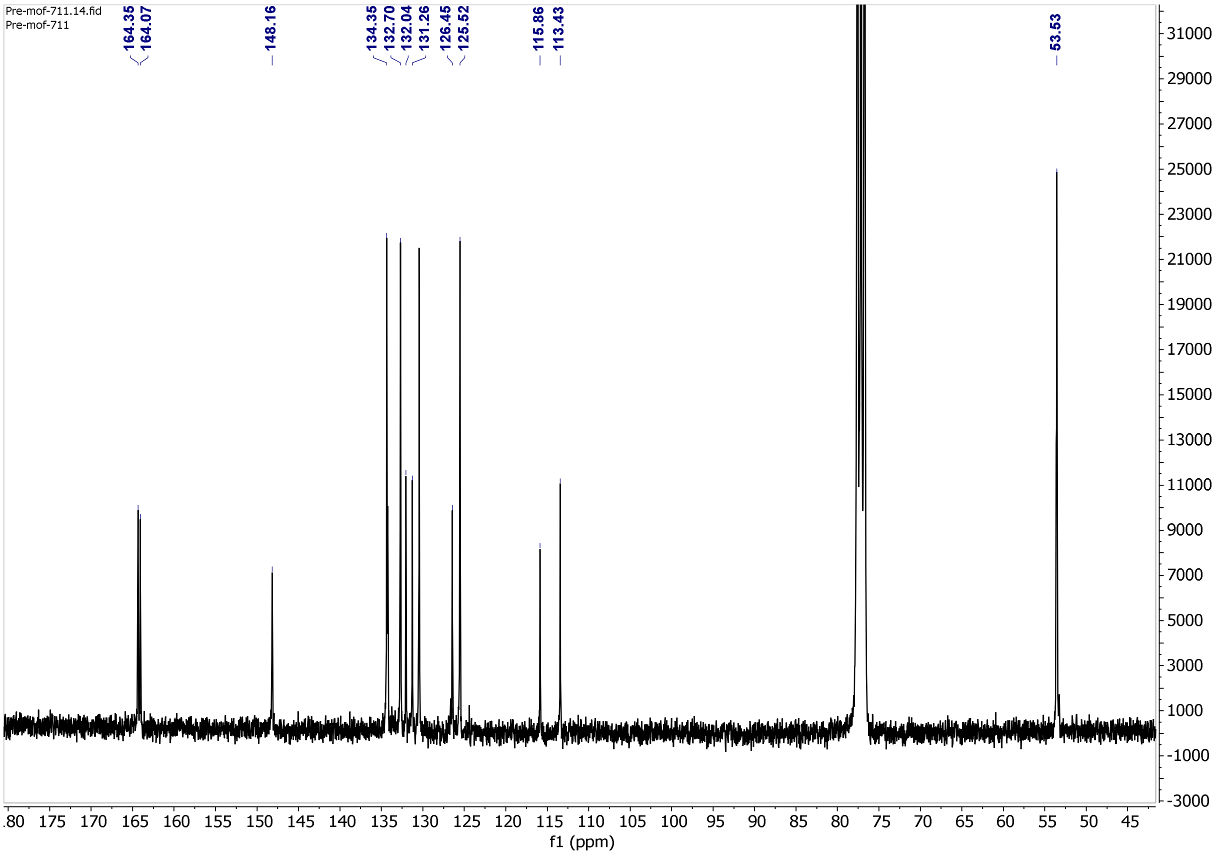

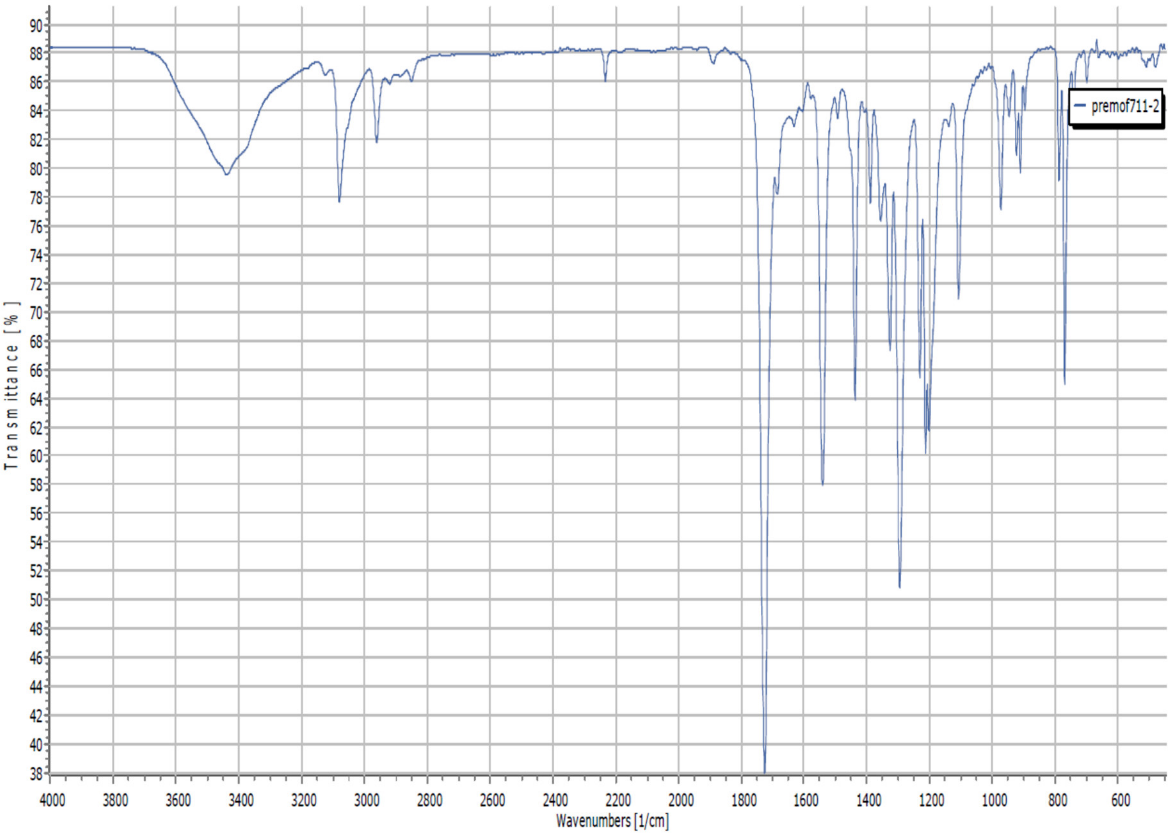

premof711-2
Transmittance [ % ]
Wavenumbers [1/cm]

Dimethyl 4-amino-8-cyanonaphthalene-2,6-dicarboxylate

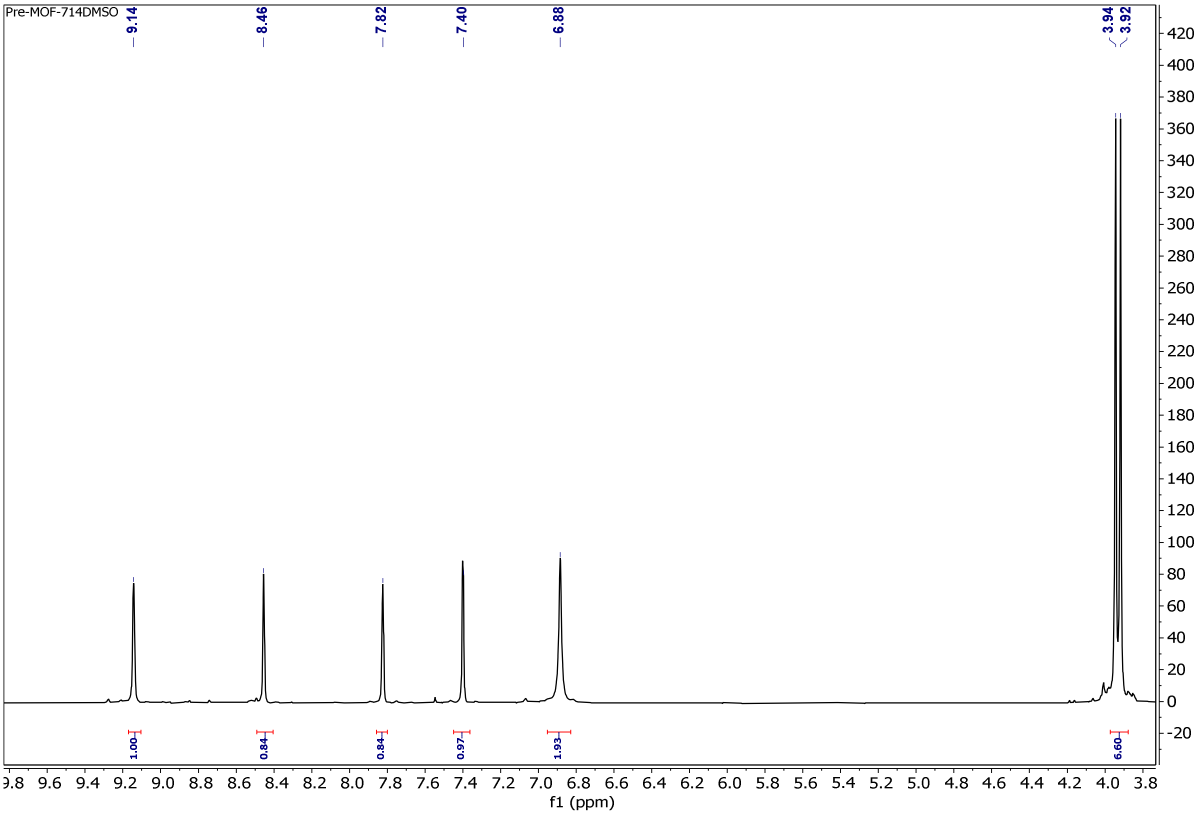


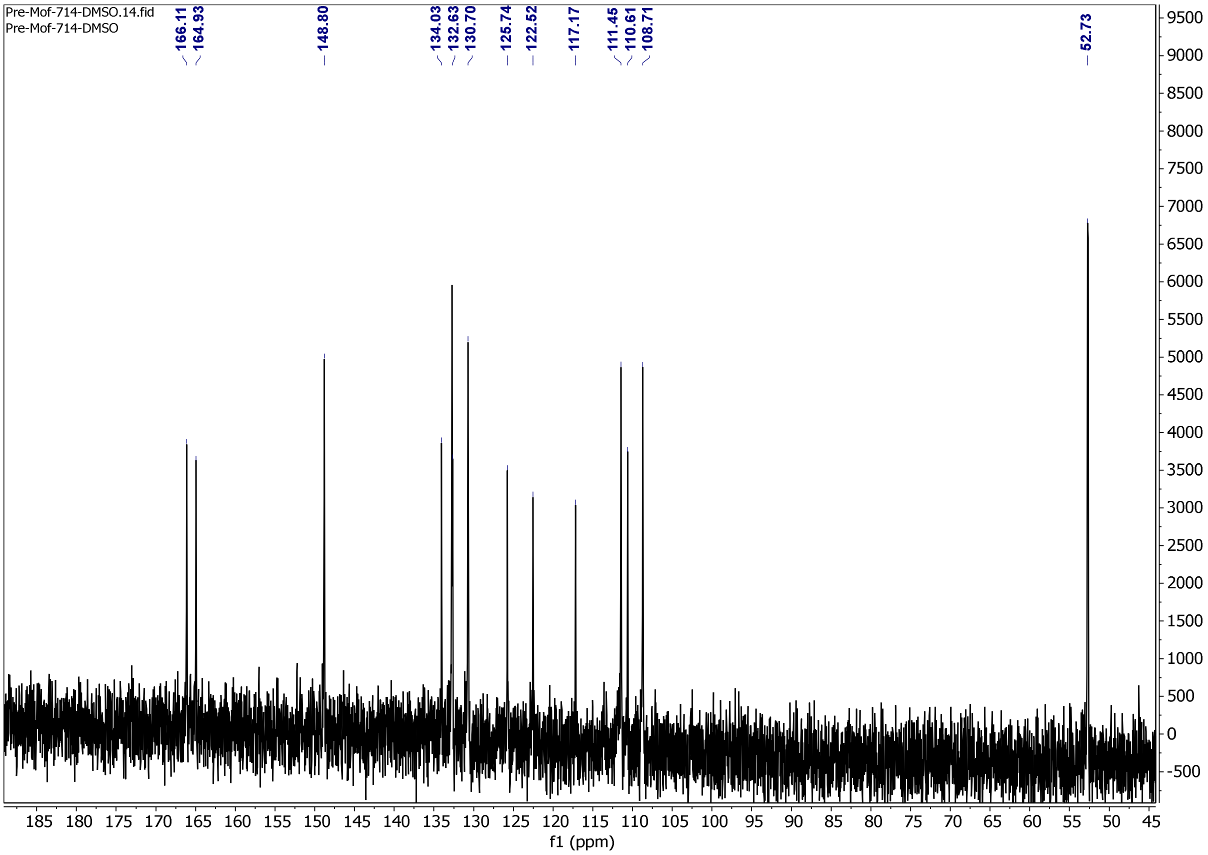

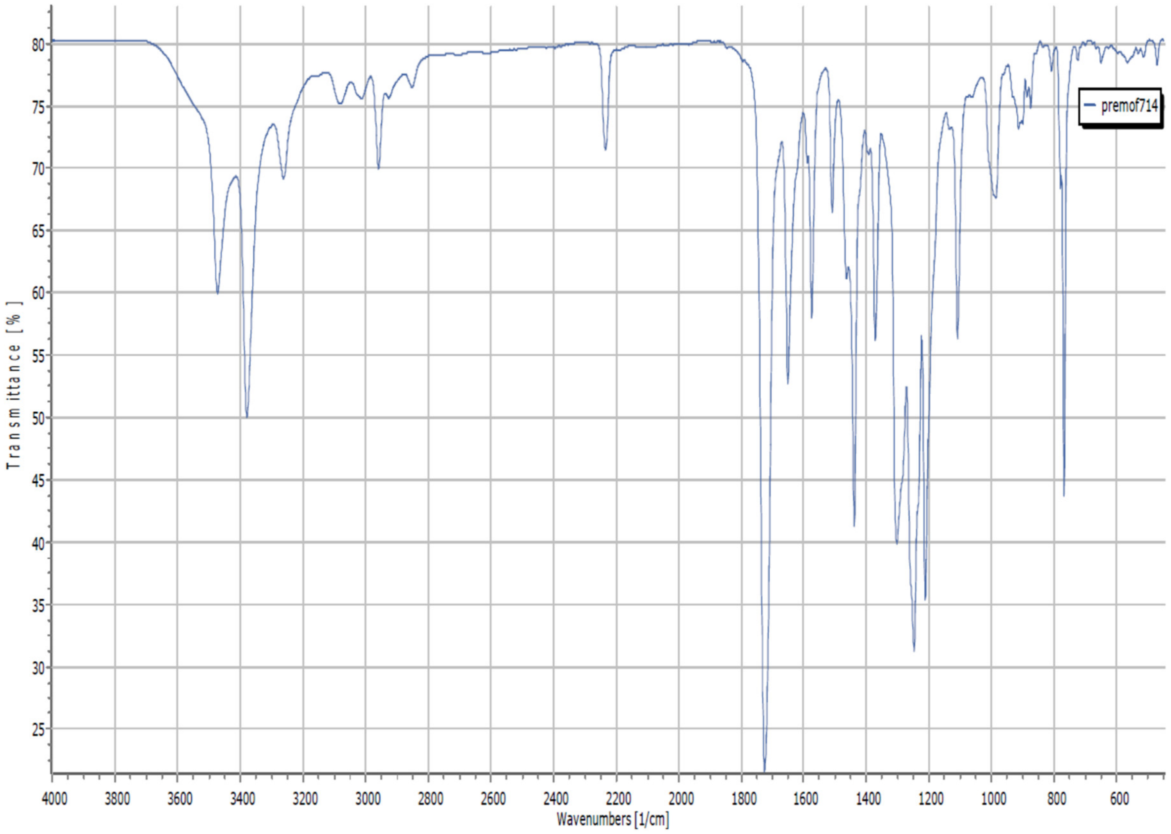

premof714
Transmittance [%]
Wavenumbers [1/cm]

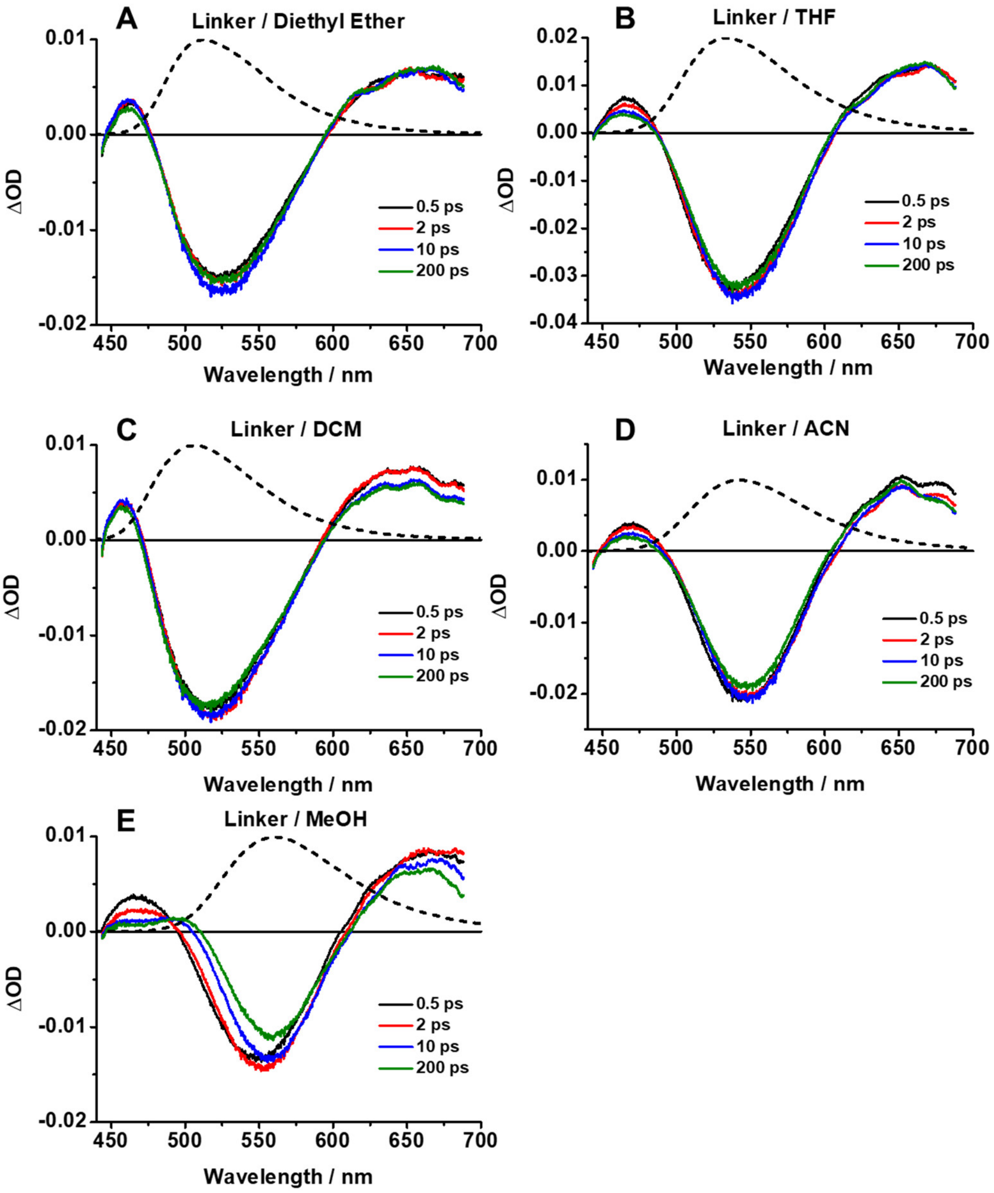


**Figure S1.** Femtosecond transient absorption spectra of $Me_2CANADC$ in (A) DE, (B) THF, (C) DCM, (D) ACN, and (E) MeOH in a 200 ps time window upon excitation at 420 nm.

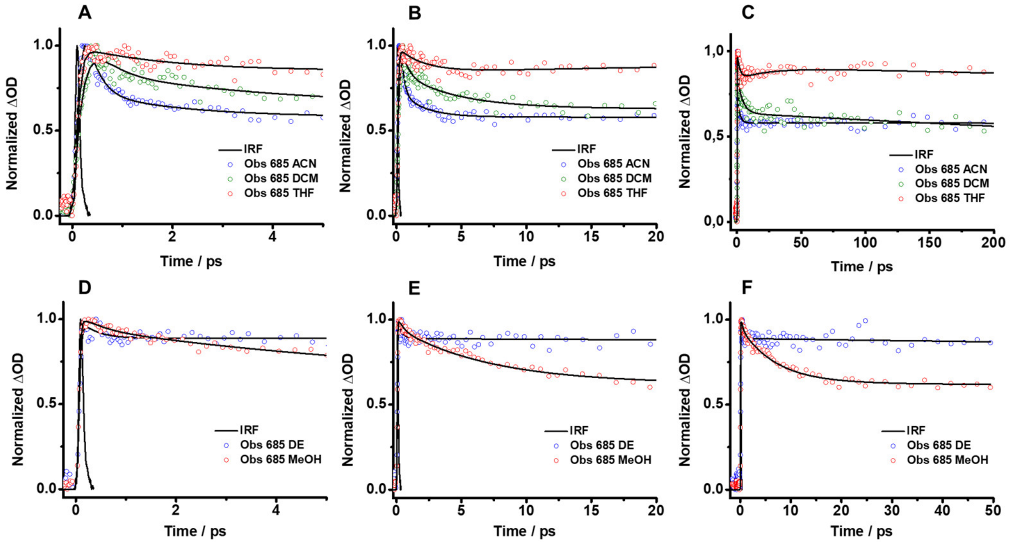


**Figure S2.** Femtosecond transient absorption decays of Me2CANADC in ACN, DCM and THF in (A) a 5 ps, (B) 20 ps and (C) 200 ps range. Femtosecond decays of Me2CANADC in DE and MeOH in (D) a 5 ps, (E) 20 ps and (F) 200 ps range, respectively. The gating wavelength was 685 nm and the samples were pumped at 420 nm.

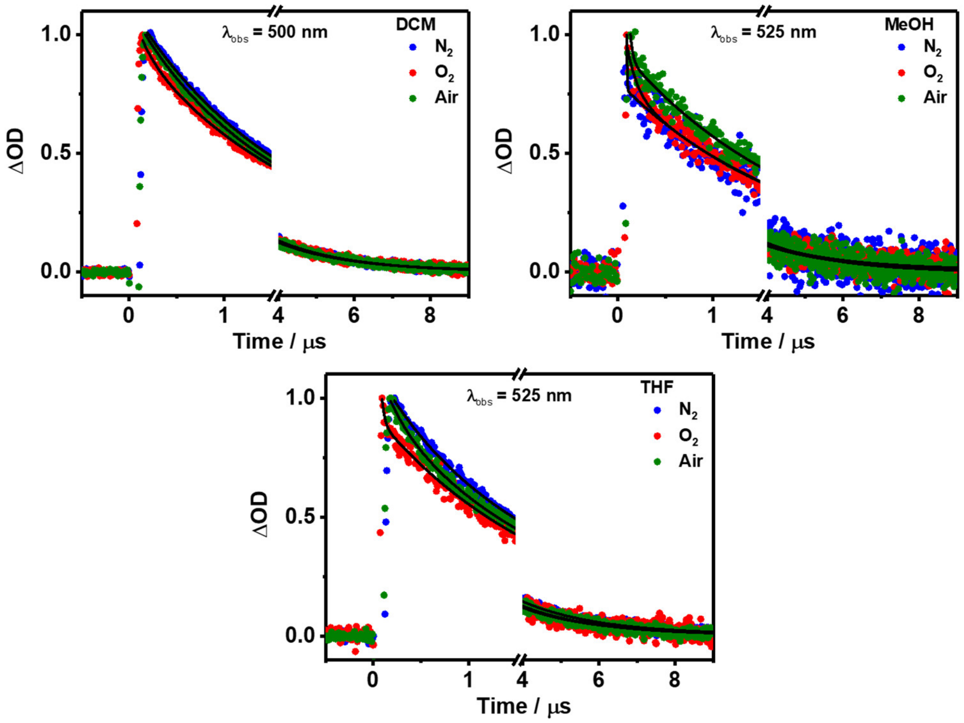


**Figure S3.** Flash photolysis transient decays of Me2CANADC under different atmospheric conditions: air (green), oxygen (red) and nitrogen (blue). The observation wavelengths were 500 nm for DCM and 525 nm for MeOH and THF. The solid black lines represent the best fit using mono or multiexponential analyses.

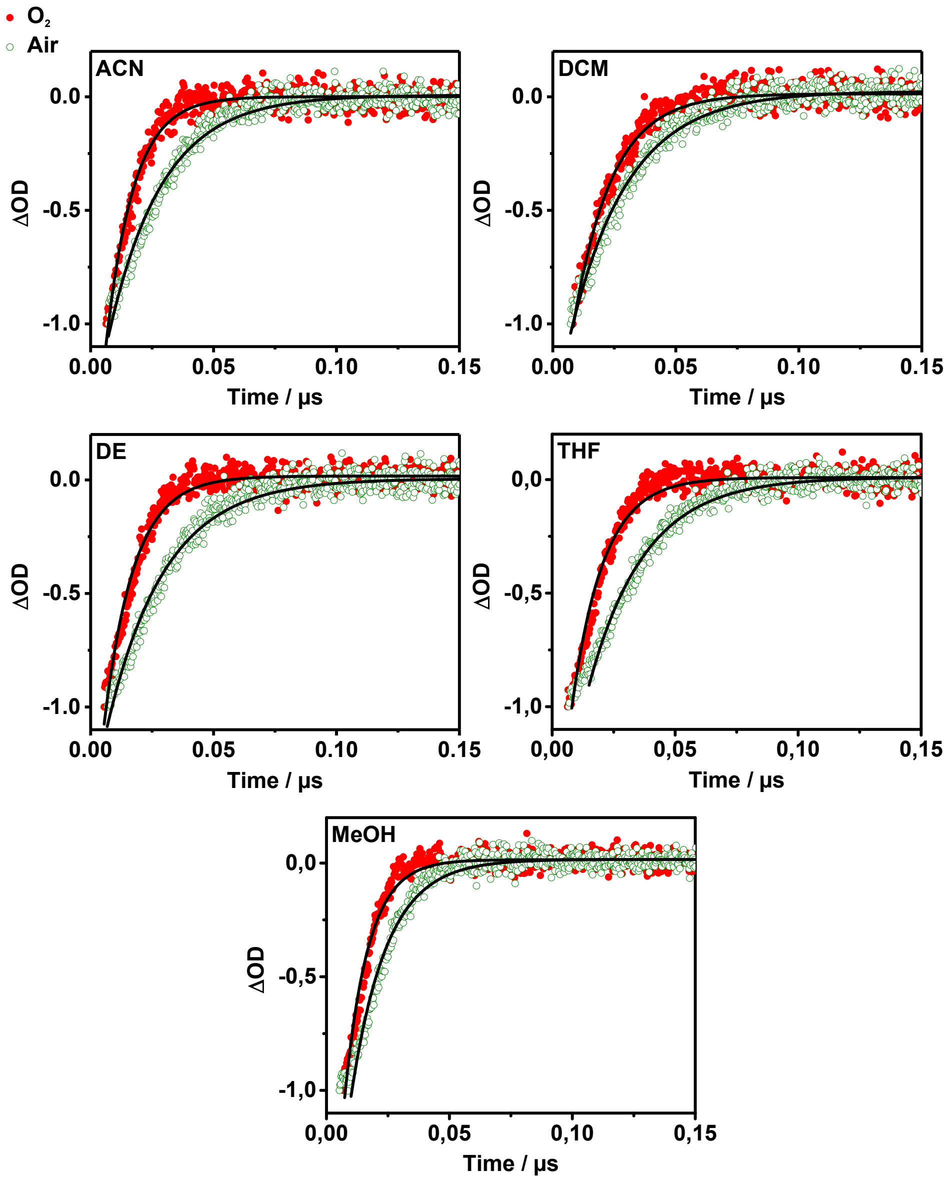


**Figure S4.** Flash photolysis transient absorption decays of $Me_2$CANADC in ACN, DCM, DE, THF and MeOH upon excitation at 420 nm at early delays in air (green) and saturated oxygen (red) atmospheres. The observation wavelength was 650 nm. The solid black lines represent the best fit using a monoexponential model.

**Table S1.** Time constants (τ), pre-exponential factors (A) extracted from the transient absorption decay fits of $Me_2$CANADC in ACN, DCM, DE, MeOH and THF upon excitation at 420 nm.

| Solvent | air | | $O_2$ | |
|---|---|---|---|---|
| | τ / ns | A | τ / ns | A |
| **DE** | **24 ± 0** | 1.45 | **12 ± 0** | 1.72 |
| **THF** | **23 ± 0** | 1.77 | **13 ± 0** | 1.89 |
| **DCM** | **24 ± 0** | 1.45 | **16 ± 0** | 1.74 |
| **ACN** | **22 ± 0** | 1.48 | **11 ± 0** | 1.91 |
| **MeOH** | **14 ± 0** | 2.08 | **10 ± 0** | 2.25 |

[i] M. Gutiérrez, R. Navarro, F. Sánchez and A. Douhal, *Phys.Chem.Chem.Phys.*,**2017**, ***19***, 16337
